# Case Report: Rescue with Impella 5.5 after HeartMate 3 failure- a bridge to heart transplantation

**DOI:** 10.3389/fcvm.2026.1947199

**Published:** 2026-10-26

**Authors:** Aarti Desai, Vrutti Patel, Andrea Muniz, Laxmi Raj Bangari, Isabel Cortopassi, Devang Sanghavi, Patricia Mergo, Ram Vivek, Devora Leventhal, John Haney, Basar Sareyyupoglu, Kevin Landolfo, Archer Martin, Parag Patel, Anna Shapiro, Rohan Goswami

**Affiliations:** 1 Department of Advanced Heart Failure and Transplant, Mayo Clinic, Jacksonville, FL, United States; 2 Department of Critical Care Medicine, Mayo Clinic, Jacksonville, FL, United States; 3 Florida State University College of Medicine, Jacksonville, FL, United States; 4 Department of Radiology, Mayo Clinic, Jacksonville, FL, United States; 5 Department of Cardiothoracic Surgery, Mayo Clinic, Jacksonville, FL, United States; 6 Department of Anesthesiology and Perioperative Medicine, Mayo Clinic, Jacksonville, FL, United States

**Keywords:** cardiogenic shock, durable left ventricular assist device, Impella 5.5, LVAD outflow graft obstruction, transplant

## Abstract

**Background:**

We present the first reported case of simultaneous HeartMate 3 (HM3) and Impella 5.5 support as a bridge to heart transplantation.

**Case presentation:**

A 35-year-old male with a history of non-ischemic cardiomyopathy presented to our facility for the evaluation of low-flow alarms 2 years post-HM3 implantation. The HM3 device was set to 5,200 RPM (2.9 L/min); the patient was asymptomatic and had no edema or orthopnea. He was actively listed for heart transplantation at the time of presentation. A chest CT scan revealed evidence of hypoattenuation next to the bend relief portion of the outflow cannula, and therefore, the patient was admitted for monitoring and transplant evaluation. Bivalirudin (0.12 mg/kg/h) was initiated. Over the next 18 days, the patient remained clinically stable with a pulsatility index ranging between 3 and 4. An echocardiogram performed on day 18 to assess clot burden revealed eccentric left ventricular hypertrophy with septum deviation to the right and a left ventricular internal diameter in diastole (LVIDd) of 70 mm, and hence, milrinone (0.25 µg/kg/min) was initiated. Right heart catheterization revealed a right atrial (RA) pressure of 18 mmHg, a right ventricle (RV) pressure of 54/2 mmHg, a pulmonary artery (PA) pressure of 55/28 (37) mmHg, a pulmonary capillary wedge pressure of 32, a pulmonary artery pulsatility index of 1.5, a Fick cardiac output of 7.7 L/min, and a Fick cardiac input of 3.4 L/min/m^2^. On day 40, the patient complained of cough, fever (38.1°C), and shortness of breath, following which he was intubated and started on meropenem (2 mg) intravenously (IV) three times a day (TID). His left ventricular assist device (LVAD) flow rate decreased to 1.2 L/min, with increasing alarm frequency and worsening hemodynamics. CT angiography revealed extensive near-occlusive hypodense filling defects in the proximal outflow cannula consistent with thrombosis, leaving only a 1–2 mm residual lumen.

The team chose to support the patient’s worsening hemodynamics and cardiogenic shock with an Impella 5.5 device rather than take him for emergent surgical exploration for his outflow graft obstruction. The Impella 5.5 device was placed in a standard fashion via the left axillary artery, and it delivered flow rates of 2.6–3.1 L/min. Concurrently, HM3 was maintained at flow rates of 1.5–2.0 L/min. Despite persistent low-flow alarms on HM3, the patient's hemodynamics improved after the Impella 5.5 device was implanted [heart rate, 101 beats per minute; BP, 104/77 (88) mmHg; RA, 2 mmHg; PA, 35/19 (24) mmHg; venous oxygen saturation (SvO_2_)], 69%), and he became ambulatory. His United Network for Organ Sharing (UNOS) status was upgraded to status 1e because of the risk of stroke. Over the next 6 days, the patient was maintained under close observation, and he remained clinically stable in the ICU. The estimated glomerular filtration rate remained between 41 and 50 mL/min/1.73 m^2^.

Six days after Impella 5.5 placement, the patient underwent a prolonged and complex heart transplant. One week post-transplant, the patient had normal biventricular graft function and stable renal function, was extubated, and was ambulatory on dobutamine medication (4 µg/kg/min). Two weeks later, the patient was discharged, with stable cardiac function and standard immunosuppression. Two months post-transplant, he showed grade 2R rejection, which was adequately treated. Currently, 6 months post-transplant, he remains stable while being maintained on the standard post-transplant immunosuppression and cardiac rehabilitation.

**Conclusion:**

This is the first reported case highlighting the feasibility of Impella 5.5 support after HM3 failure as a bridge to transplantation for short-term hemodynamic stabilization in the setting of LVAD outflow obstruction and cardiogenic shock without the need for extracorporeal membrane oxygenation or emergent chest exploration, which may complicate or delay transplantation.

## 1 Background

Recent estimates suggest that nearly 500,000 new cases of heart failure are diagnosed annually in the United States, highlighting the growing burden of advanced heart disease (1). As transplantation is limited by donor availability, the use of mechanical circulatory support (MCS) devices—temporary and durable—is on the rise.

### 1.1 MCS devices: HeartMate 3 and Impella 5.5

HeartMate 3 (HM3) is a third-generation, continuous-flow left ventricular assist device (LVAD) designed for patients with end-stage left ventricular heart failure as destination therapy or as a bridge to transplantation. HM3 is a magnetically levitated centrifugal-flow pump designed for long-term support and demonstrates markedly reduced rates of pump thrombosis compared with earlier generations of LVADs (0 and 1.1% at 6- and 24-month postimplantation, respectively) (2).

Complications associated with HM3 are often categorized as device dysfunctions and patient events. The MOMENTUM trial and the results published from the Manufacturer and User Facility Device Experience (MAUDE) Database revealed that among device events, flow disruption was the most common (21%), followed by halted pump (9.2%) and material deformation (8.4%) (3, 4). The same trial found that in patient adverse events, the most common effects were blood loss (21.8%), infection (17.8%), and thrombus (3.3%) (3). Other adverse compilations include pump thrombosis and outflow graft obstruction (OGO). Although the risk of thrombosis, as with any implant, is considerable, there has been a vast improvement in the HM3 device when compared it with its previous generations (0 and 1.1% at 6- and 24-month postimplantation, respectively) (2). The rates of OGO vary in studies, ranging from 1% to 7%, with a multicenter trial by Wert et al. showing an OGO incidence of 3.0% (5, 6). Emerging studies have found that biodebris (proteinaceous substance) accumulation between the outflow graft and the bend relief portion is another common extrinsic cause of obstruction in patients with the new HM3 device, resulting in significant morbidity and mortality (7).

The Impella 5.5 device is a minimally invasive microaxial catheter surgically placed across the aortic valve and is used for short-term shock stabilization (8). Unlike veno-arterial extracorporeal membrane oxygenation (VA-ECMO), Impella 5.5 does not increase the left ventricular afterload (9). In fact, there is sufficient evidence that demonstrates enhanced LV unloading, with Impella 5.5 improving renal and hepatic perfusion compared with other modes, suggesting enhanced end-organ perfusion (10, 11). Complications include bleeding, thrombosis, vascular access complications, and infection (12).

### 1.2 Concurrent use of both devices

The concurrent use of a durable LVAD and Impella 5.5 has not been documented. Although concurrent multidevice support is associated with increased complication rates, Impella 5.5 remains a rescue device for many such configurations. A well-known use of Impella 5.5 for the purpose of left ventricular unloading while on ECMO support is known as EcPella (13). Another case series of four patients described the combined use of Impella 5.5 and ProtekDuo to achieve right ventricular unloading and pulmonary decongestion and reduce the use of pressors (14).

Herein, we present the first reported case where the Impella 5.5 support device was used to stabilize hemodynamics in a patient with a durable LVAD outflow graft obstruction as a bridge to transplantation.

## 2 Case presentation

A 35-year-old male with a history of non-ischemic cardiomyopathy status post-LVAD (HM3), tricuspid valve repair before 2.5 years, chronic kidney disease [estimated glomerular filtration rate (eGFR), 51 mL/min/1.73 m^2^], hypertension, and diabetes, but with no history of coagulation disorders, presented to our facility for the evaluation of low-flow alarms. A few months prior to the HM3 implantation, he suffered a middle cerebral artery stroke that resulted in residual right-sided weakness and left ventricular thrombus that was treated with apixaban (5 mg). The immediate post–HM3 period was complicated by RV failure requiring a right ventricular assist device for 6 days. At-home medications included 81 mg of aspirin once daily (OD), 100 mg of hydralazine four times a day (QID), 60 mg of nifedipine twice daily (BID), 24/26 mg of Entresto BID, 20 mg of torsemide OD, and 6 mg of warfarin OD [international normalized ratio (INR), 2.6]. The patient was asymptomatic, without edema or orthopnea. HM3 was set to 5,200 RPM, and it had a flow rate of 2.9 L/min and a power consumption of 3.5 W.

An echocardiogram that was taken 15 days prior to admission showed a left ventricular internal diameter in diastole (LVIDd) of 64 mm, a poorly visualized RV but with observable reduced function, and no visualization of the outflow graft. After admission, a chest CT scan was performed, which revealed evidence of hypoattenuation next to the bend relief portion of the outflow cannula, and therefore, the patient was admitted for monitoring and transplant evaluation. Bivalirudin (0.12 mg/kg/h) was initiated during inpatient admission. Over the next 18 days, the patient remained clinically stable, the hemoglobin level was 10.7 g/dL, hematocrit was 32.9, the activated partial thromboplastin time (aPTT) was 57–90 s, and the pulsatility index remained between 3 and 4. Another echocardiogram performed on day 18 of admission to assess clot burden revealed an LVIDd of 70 mm, eccentric LV hypertrophy with septum deviation to the right, and reduced right ventricular function. The aortic valve (AV) opened for every cycle. Milrinone (0.375 µg/kg/min) was initiated. Right heart catheterization with milrinone support revealed a right atrial (RA) pressure of 18 mmHg, a right ventricle (RV) pressure of 54/2 mmHg, a pulmonary artery (PA) pressure of 55/28 mmHg (mean: 37 mmHg), a pulmonary capillary wedge pressure (PCWP) of 32 mmHg, a pulmonary artery pulsatility index (PAPi) of 1.5, an SpO_2_ of 95%, a Fick cardiac output (CO) of 7.7 L/min, and a Fick cardiac index (CI) of 3.4 L/min/m^2^, and they remained unchanged.

On day 40, he complained of cough, fever (38.1°C), and shortness of breath (SOB). Imaging confirmed left-sided pneumonia. He was intubated and started on meropenem (2 g) intravenously (IV) three times a day (TID). Outflow obstruction worsened, with LVAD flow rates reducing to 1.2 L/min (5,200 RPM, 3.3 W) with increased alarm frequency (>20 times/day), the blood pressure was 80–92/50–62 mmHg, and the mean arterial pressure (MAP) ranged between 55 and 62 mmHg. CT angiography revealed extensive near-occlusive hypodense filling defects in the proximal outflow cannula consistent with thrombosis, leaving only a 1–2 mm residual lumen (Figure 1). INR remained between 1.4 and 2.0 and lactate dehydrogenase (LDH) levels between 184 and 211 U/L, while the hemoglobin level was low (between 8.3 and 9.4 g/dL) in the prior week.

**Figure 1:**
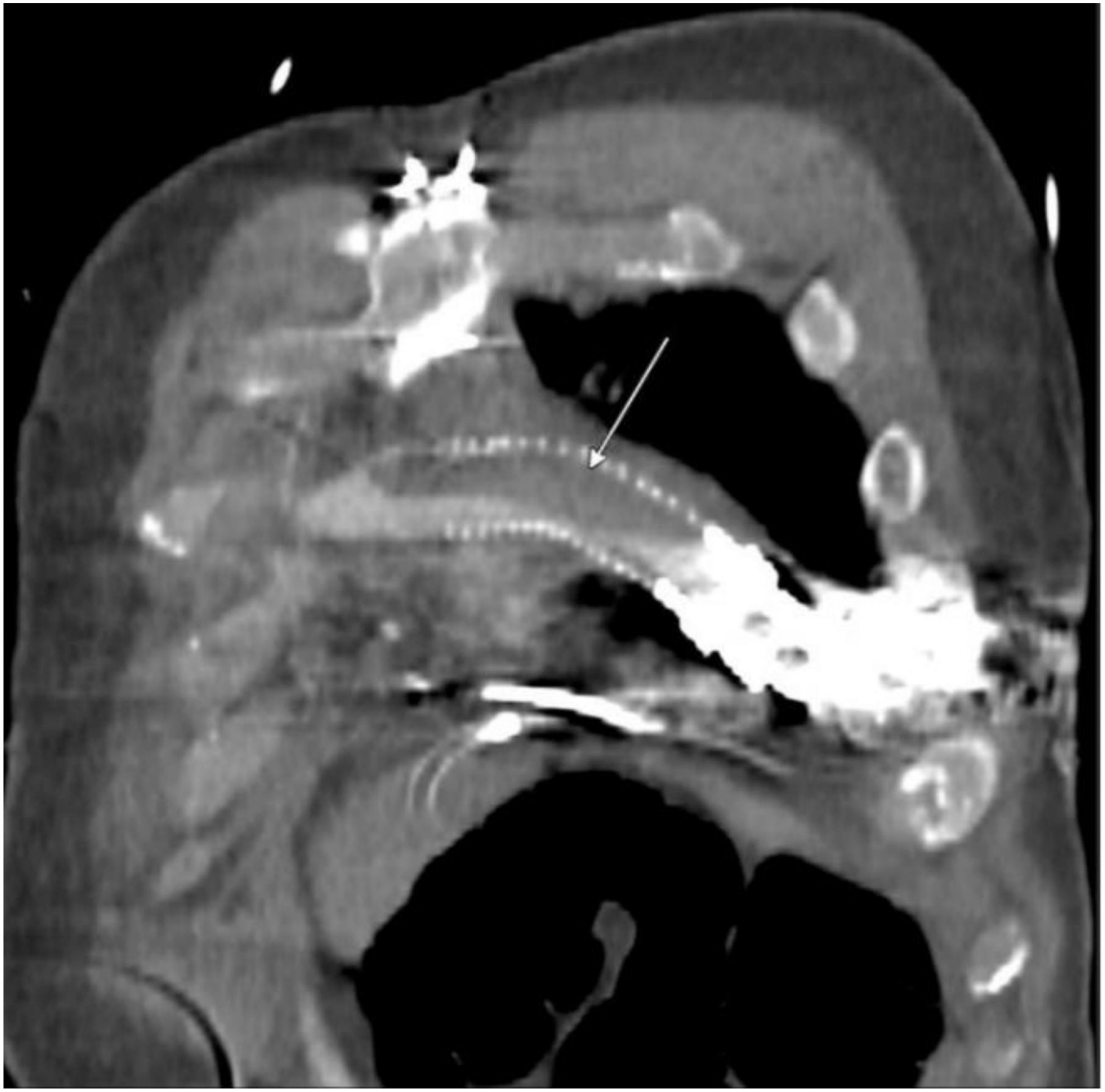
A coronal reformatted image of a chest CT angiography reveals an extensive hypodense filling defect in the proximal outflow cannula of the HeartMate 3 LVAD, consistent with near-occlusive thrombus leaving only a 1–2 mm residual lumen.

After multidisciplinary discussions, the use of extracorporeal membrane oxygenation was deemed inappropriate because of his left ventricular hypertrophy, and the LVIDd was 70 mm. In addition, increased LV afterload in a patient with pulmonary hypertension and limited mobility awaiting transplant favored temporary mechanical circulatory support (tMCS) over ECMO. The team moved to support worsening hemodynamics and shock with the Impella 5.5 tMCS device. Impella 5.5 was inserted via a surgical graft into the left axillary artery. After ensuring proper positioning, the Impella 5.5 flow was initiated slowly and set to P5, delivering flow rates of 2.6–3.1 L/min (Figure 2) while simultaneously decreasing HM3 flow rates to 1.5–2.0 L/min to ensure a balanced flow. There were no intraoperative complications. Despite persistent low-flow alarms on HM3, hemodynamics improved after Impella 5.5 was implanted [HR, 101 beats per minute (BPM); BP, 104/77 (88) mmHg; RA, 2 mmHg; PA, 35/19 (24) mmHg; SvO_2_, 69%], and the patient became ambulatory under the guidance of our physiotherapists and occupational therapists.

**Figure 2:**
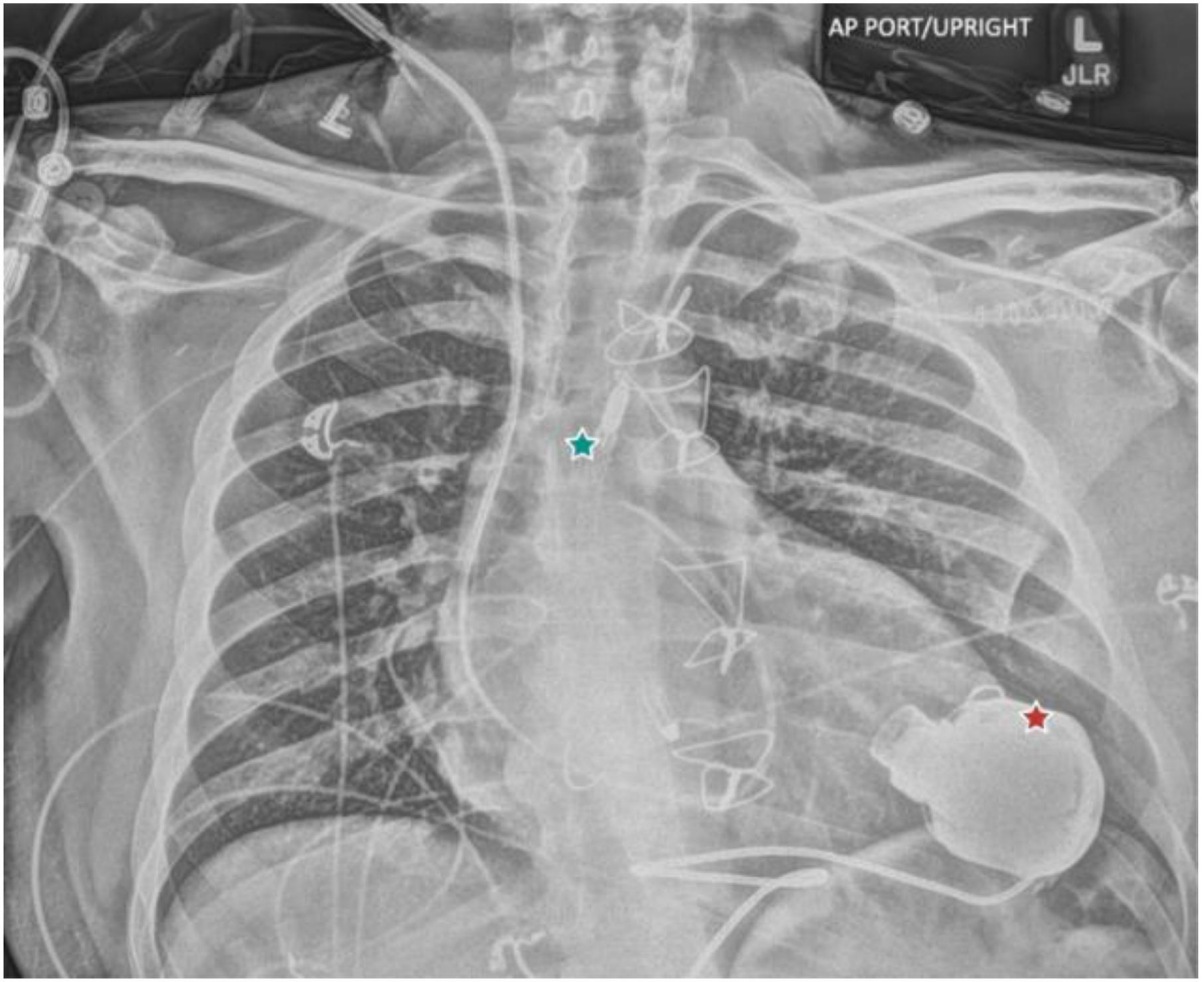
A frontal chest X-ray showing HeartMate 3 (red star) and Impella 5.5 (green star) inserted via the left axillary artery.

The patient’s United Network for Organ Sharing (UNOS) status was upgraded to status 1e because of the risk of stroke. Over the next 6 days, the patient was under close observation, and he remained clinically stable in the ICU. eGFR remained between 41 and 50 mL/min/1.73 m^2^. The patient remained on dual HM3 + Impella 5.5 support for 6 days, and during this time, he received a successful donor match.

The transplant was complex and prolonged (>12 h OR time). The outflow graft of HM3 densely adhered to the surrounding structures and required careful dissection. Explant pathology results showed a layered thrombus within the graft. The transplant required 10 units of packed RBCs (pRBCs), and vasoplegia was managed with dobutamine (5 µg/kg/min) and vasopressin (0.04 U/h). On postoperative day (POD) 1, the patient underwent a mediastinal exploration and hematoma evacuation. By POD 6, he was weaned off inotropes, had normal biventricular graft function and stable renal function, was extubated, and became ambulatory on dobutamine (4 µg/kg/min). He was discharged on POD 15, with stable cardiac function, and he was given tacrolimus (6 mg) BID and mycophenolate (500 mg) BID for immunosuppression.

Currently, six months post-transplant, he remains stable while also receiving standard post-transplant immunosuppression medication, prophylaxis, and outpatient cardiac rehabilitation.

## 3 Discussion

This is the first reported case of the use of Impella 5.5 for short-term hemodynamic stabilization in a patient with LVAD OGO as a bridge to transplantation. With this in mind, we felt that it was important to discuss our rationale behind device selection and the unique challenges of managing concurrent device support.

### 3.1 Evaluation and management of low-flow alarms and outflow graft obstruction

Our patient presented to the facility for the evaluation of low-flow alarms that suggested LVAD OGO. The obstruction may be mechanical such as a kink in the outflow graft, positional, due to low-volume states or most commonly, thrombosis. Pump thrombosis, one of the complications associated with LVAD OGO, is defined as any clot formation in the flow path of the pump. It may lead to stroke, peripheral thromboembolism, and, in severe cases, pump failure, leading to worsening shock (15).

Multimodality evaluation should be conducted to fully understand the cause of the obstruction using laboratory values (LDH, plasma free hemoglobin, indirect bilirubin, haptoglobin, and renal function), an echocardiogram, and radiographic imaging techniques such as a CT angiogram (16). In our patient, the CT angiogram (Figure 1) confirmed the diagnosis by showing extensive thrombosis despite adequate anticoagulation with warfarin and then bivalirudin. In patients with impaired renal function, contrast-induced renal injury should be considered before performing CT angiography. In such cases, imaging techniques such as a transesophageal echocardiogram and right heart catheterization may be used to drive diagnosis, in addition to a comprehensive review of laboratory values mentioned above. In specialized centers, directed angiography of the outflow graft via retrograde fashion may allow for limited contrast injection.

Management usually involves a combination of intensified anticoagulation and pump replacement, with the latter being definitive treatment (17). A systematic review by Dang et al. found that in patients with no or partial resolution of pump thrombi, thrombolytic regimens and non-thrombolytic regimens had no significant differences overall [odds ratio (OR), 0.48; 95% confidence interval (CI), 0.20–1.16] (18). The decision to forego surgical management options such as evacuation via thoracotomy, endovascular stenting, and balloon angioplasty was taken because of the extensive nature of the clot resulting in near-zero flow and the need to further support the left ventricle (19, 20).

### 3.2 Impella 5.5 placement and intraoperative management

The decision for Impella 5.5 implantation over other options for hemodynamic stabilization required multidisciplinary discussion among the critical care, anesthesia, and transplant teams. The factors that weighed into the decision included coordinating with the existing LVAD cannula, balancing flow between the two devices, and methods of placement.

Although ECMO was being considered to improve oxygenation, the inherently non-physiological profile of VA-ECMO—often causing retrograde aortic flow that increases left ventricular afterload, which may worsen ventricular distension and pulmonary edema—led to the decision to opt against it. In addition, at presentation, our patient had LV distension (LVIDd, 70 mm), LV hypertrophy, and septal deviation toward the right ventricle, increasing the probability of worsening of LV function post–ECMO placement. This, in conjunction with the more physiological hemodynamic profile of Impella 5.5 such as enhanced LV unloading, preserved end-organ perfusion (particularly renal perfusion), biventricular function recovery, and pulmonary decongestion, made it the safer option (11, 21, 22).

### 3.3 Dual-device interaction and complex flow dynamics

The feasibility of Impella insertion with failing LVAD, such as HeartMate 3 thrombosis or outflow graft obstruction, was of paramount importance in our patient. While considering the implications of simultaneous usage, the primary concern, although theorized, was aortic valve damage from both the effects on flow dynamics at the aortic root and the local trauma due to the placement of the Impella 5.5 device. Other theorized risks of combined support include abnormal flow dynamics such as the risk of recirculation (re-entry of blood through an open aortic valve caused by higher pressures at the aortic root), hemolysis, and increased thrombus risk. As this is the first reported case of concurrent support, the safety profile remains unclear, and further investigation is warranted before dual-device management strategies can be recommended.

### 3.4 Heart transplant and intraoperative management

Orthotopic heart transplantation (OHT) after durable LVAD support is inherently complex due to adhesions, altered mediastinal anatomy, and hemodynamic challenges associated with the device. In our patient, the presence of a densely adherent outflow graft with near-occlusive thrombosis further increased the surgical complexity, necessitating prolonged dissection and cardiopulmonary bypass time. The patient required significant blood transfusion support and postoperative hematoma evacuation, consistent with the known increased bleeding risk in this population. Despite these intraoperative and early postoperative challenges, the patient demonstrated favorable graft function and recovery, underscoring the importance of careful perioperative planning, multidisciplinary care, and the availability of advanced surgical expertise in high-volume transplant centers. This case also highlights that, even in technically demanding scenarios, timely transplantation remains the definitive therapy for patients with refractory LVAD complications.

### 3.5 Updates in LVAD thrombosis management

The evolution of third-generation devices such as HeartMate 3 has significantly reduced the incidence of pump thrombosis compared with earlier LVAD platforms. Consequently, the clinical focus has shifted toward managing bleeding complications, which are frequently attributed to gastrointestinal arteriovenous malformations and acquired von Willebrand factor deficiency (23). An area of ongoing discussion in antiplatelet management involves determining the optimal aspirin dosing strategy. The MOMENTUM-3 trial showed that there was no significant difference between 81 mg of aspirin and 325 mg of aspirin in HM3-supported patients with regard to bleeding and thrombotic risks (24). There is a lack of studies in the literature on whether it can be completely stopped in lieu of newer anticoagulants. Trials are underway to assess the efficacy of low-intensity anticoagulation while also aiming to reduce gastrointestinal (GI) bleeding and other bleeding complications (25).

Our case illustrates that even with adequate anticoagulation using warfarin and parenteral bivalirudin, clinically significant outflow graft thrombosis can still occur in HM3. This underscores the significance of vigilant surveillance, the role of multimodality imaging in early detection, and the need for novel approaches for the prevention and management of both thrombotic and hemorrhagic complications in LVAD-supported patients.

## 4 Conclusions

This case demonstrates the feasibility of Impella 5.5 support in the setting of HeartMate 3 outflow graft obstruction as a bridge to transplantation. By providing short-term hemodynamic stabilization and effective ventricular unloading, Impella 5.5 served as an effective alternative when ECMO was contraindicated. Dual-device support required meticulous hemodynamic management but allowed the patient to remain stable and ambulatory until a suitable donor heart became available. This experience highlights the importance of individualized, multidisciplinary decision-making in managing complex LVAD complications and suggests that Impella 5.5 may represent a safe and effective rescue strategy in select patients awaiting heart transplantation.

## Data Availability

The raw data supporting the conclusions of this article will be made available by the authors, without undue reservation.
